# AI-Aided Crystallization Elution Fractionation (CEF) Assessment of Polyolefin Resins

**DOI:** 10.3390/polym17121597

**Published:** 2025-06-07

**Authors:** Lorenzo Brighel, Gabriella Maria Lucia Scuotto, Giuseppe Antinucci, Roberta Cipullo, Vincenzo Busico

**Affiliations:** 1 Department of Chemical Sciences, Federico II University of Naples, via Cinthia, 80126 Napoli, Italy; l.brighel@studenti.unina.it (L.B.); gab.scuotto@studenti.unina.it (G.M.L.S.); giuseppe.antinucci@unina.it (G.A.); rcipullo@unina.it (R.C.); 2 Dutch Polymer Institute, 5600 AX Eindhoven, The Netherlands

**Keywords:** crystallization elution fractionation, artificial intelligence, machine learning, polyolefin characterization, mechanical recycling

## Abstract

Artificial Intelligence (AI) tools and methods are dramatically innovating the application protocols of most polymer characterization techniques. In this paper, we demonstrate that, with the aid of custom-made and properly trained machine learning algorithms, analytical Crystallization Elution Fractionation (aCEF) can be changed from an ancillary to a standalone approach usable to identify and categorize commercially relevant polyolefin materials without any prior information. The proposed protocols are fully operational for monomaterials, whereas for multimaterials, integration with AI-aided ^13^C NMR is a realistic intermediate step.

## 1. Introduction

The spread of Artificial Intelligence (AI) is revolutionizing—inter alia—the analytical approach to polymeric materials. Ultrafast classification and property evaluation methods are essential for applications in high-throughput experimentation and to sort post-consumer mixtures for recycling purposes [1,2,3,4]. In the latter domain, rapid automated identifications and separations of the various components in multimaterial waste streams, primarily based on Near-Infrared (NIR) spectroscopic fingerprinting [5,6,7,8], are now fully operational, and for several polymer classes (e.g., polyesters and polyamides), the fraction of recycled products is close to that of paper, glass, and some common metals [9,10]. A most notable exception is represented by polyolefins (POs), because the lack of idiosyncratic functional groups in the polymer chains complicates the NIR categorization of the various polyethylene (PE) and polypropylene (PP) grades [1,8]. Unfortunately, these amount to about 50% by weight of the polymer market [11,12,13], and are very difficult to re-use in mixtures because thermodynamic incompatibility results in phase separation issues [14].

^13^C NMR is the only spectroscopy potentially able to finely unravel the microstructural details of PO blends [15,16,17,18]. Compared with NIR, ^13^C NMR is a much more demanding technique in terms of Capex and Opex. On the other hand, the introduction of high-temperature cryoprobes has greatly reduced the time for the acquisition of quantitative PO spectra (from several hours down to a few minutes) [16,17,18]; therefore, a spectrometer equipped with one such probe can process up to several hundred samples per day. In ref. [15], we introduced AI methods capable of automating ^13^C NMR PO data analysis downstream of spectral acquisition; applications can open the door to higher-value-added solutions for mechanical recycling, because a previous microstructural assessment enables the rational design of compatibilizers’ packages [19,20,21,22].

**Figure 1:**
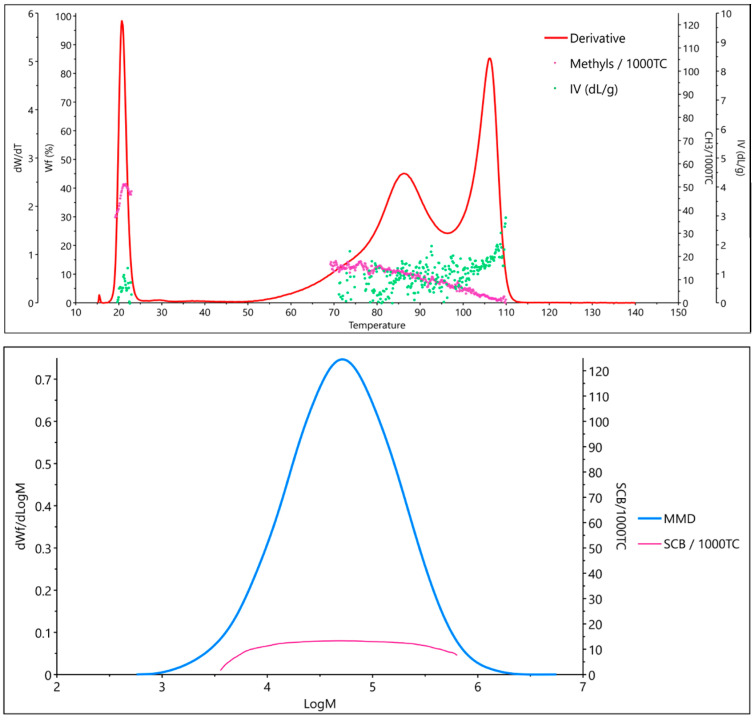
Characterization of a typical LDPE sample by aCEF (**top**) and GPC (**bottom**).

The integration of ^13^C NMR with average molar mass information would represent a further important step forward. To the best of our knowledge, hyphenating NMR spectroscopy with Gel Permeation Chromatography (GPC) has been achieved successfully (albeit not commercially) for ^1^H NMR, but not yet for ^13^C NMR [23,24]. Moreover, GPC curves of PO mixtures are poorly resolved and therefore difficult to analyze. A potentially better option is to integrate ^13^C NMR with analytical Crystallization Elution Fractionation (aCEF) data [25,26]. The aCEF technique was introduced to determine the distribution of crystallizable sequences in a PO sample from its solvent elution profile in a temperature ramp, but modern aCEF equipment can also provide the intrinsic viscosity of the material at each point of the elution curve [25,27]; moreover, the resolution is superior to GPC (Figure 1).

In the present paper, we demonstrate that machine learning methods can be implemented to categorize a PO material *based on aCEF information alone*. This is an important first achievement for the automated application of this technique in the realm of fast polyolefin analytics. The approach readily applies to monomaterials, whereas extension to multimaterials is feasible, but complex, and requires further work, because the contributions from the individual components of a PO mixture to the overall aCEF trace are not necessarily additive due to cocrystallization effects. Along with covariance issues associated with limitations in resolution, this calls for a more sophisticated training of the machine learning algorithm; we will elaborate on this question in the final section.

## 2. Materials and Methods

### 2.1. Dataset Description and Pre-Processing

In this study, a diverse set of PO monomaterials was analyzed by aCEF to develop a machine learning pipeline for recognition and classification purposes from the elution profiles. For PO sample classification, we adopted the scheme proposed in Ref. [15] (Supporting Information (SI), Table S1). The monomaterial constraint was essential to accurately establish the baseline analytical behavior and elution characteristics of each PO class without confounding effects due to overlapping or interacting signals from multiple classes.

The data were obtained from the output files generated by a Polymer Char aCEF [28] setup, which, for each given sample, provides the elution profile hyphenated with the point-by-point signals from a FTIR detector (in a limited spectral region transparent to the eluent, and yet usable to determine the ratio between methyl and total carbon atoms), and a dual capillary viscometry detector providing the value of intrinsic viscosity.

Four dedicated Python scripts were implemented to automate the analysis pipeline, each addressing a specific stage of the data workflow. The first script processes raw output files from the Polymer Char aCEF measurement system and reorganizes them into structured Excel spreadsheets tailored for downstream analysis. For each PO (sub-)class, the script extracts key analytical parameters directly from the aCEF traces, including:

- The elution signal profile (“Derivative Norm”);
- The ratio between methyl and total carbon atoms;
- The intrinsic viscosity.

The elution signal profile provides a detailed distribution of crystallinity within the sample. The ratio between methyl and total carbon atoms is an essential microstructural marker for quantifying short-chain branching, and can be used to distinguish, e.g., HDPE from LLDPE and iPP class. Moreover, intrinsic viscosity serves as an indirect measure of molecular weight distribution. The combination of these parameters can give information about the composition, polymer microstructure, and crystallinity of the polymer materials that contribute to its identification and categorization.

In addition, the script integrates external metadata files that contain sample identifiers and comonomer composition in case of copolymers; this is critical for determining the specific makeup of each sample (SI, Table S2). To ensure data integrity, a filtering step was implemented to exclude any samples lacking the aforementioned data.

### 2.2. Data Processing Pipeline

The core of the analysis is an automated, Python-based (ver. 3.11.8) data processing pipeline specifically developed for the comprehensive analysis of elution peaks in aCEF traces of PO samples. This custom script handles the complete analytical workflow, from importing pre-processed elution data to generating structured descriptors of the elution signal that are optimized for statistical interpretation and machine learning tasks. The pipeline is tailored to the unique characteristics of PO aCEF data and integrates advanced signal processing, robust peak detection, and physically meaningful parametric fitting.

The process begins by importing the elution traces previously generated during the initial pre-processing stage. Each trace undergoes a smoothing operation based on spline interpolation [29], which effectively reduces high-frequency noise and baseline fluctuations while preserving the shape, asymmetry, and subtle features of the elution peaks. This step is critical, as accurate identification and modeling of elution features depend on the integrity of these signals. After smoothing, the traces are normalized with respect to their total integral, ensuring comparability across samples by providing a unified intensity scale.

Following normalization, a custom elution peak detection function is applied. This algorithm locates local maxima based on the first derivative of the smoothed signal and enforces dynamic thresholding criteria involving peak prominence and local peak-to-baseline contrast. Only those peaks that satisfy strict significance conditions, indicative of true elution contributions, are retained. This selection step is essential, especially for samples containing overlapping or noisy signals, where artifacts or ‘micropeaks’ might otherwise contaminate the descriptor space and reduce the reliability of downstream analyses.

For each selected elution peak, a non-linear least-squares fitting procedure is performed using a linear combination of Exponentially Modified Gaussian (EMG) functions. The EMG model is particularly well-suited for describing aCEF peaks, as it captures the asymmetric tailing effects resulting from mass transport and other diffusion related phenomena during sample elution. Each EMG is defined by four parameters (Equation (1)), namely amplitude (A), center (c), standard deviation (σ), and exponential decay constant (τ), allowing for a flexible, yet physically interpretable, reconstruction of the elution peak shape.

(1) $$EMG\left(x\right)=A\exp\left(\frac{1}{2}{\left(\frac{\sigma }{\tau }\right)}^{2}-\frac{x-c}{\tau }\right)\mathrm{erfc}\left(\frac{1}{\sqrt{2}}\left(\frac{\sigma }{\tau }\right)\right)$$

The fitting process uses bounded minimization routines under constraints that enforce physical realism and avoid overfitting.

Fit accuracy is validated by comparing the area under the combined EMG curve to the corresponding segment of the experimental trace. A fit is accepted if it captures at least 90% of the local area under the curve in the elution peak region. If this criterion is not met, an iterative fitting improvement process is initiated: at each iteration, peak detection is repeated with an increased minimum standard deviation requirement, effectively filtering out noise and allowing for additional EMG components to be added only when justified. This refinement continues until the accuracy threshold is achieved and no significant residuals remain (SI, Figure S1).

Once the fitting process is completed, a set of quantitative descriptors is extracted for each EMG component in the elution trace. These include shape parameters, peak area, and structural features, such as methyl per thousand carbon atoms and average intrinsic viscosity measured at the peak location. The resulting descriptors are saved on an Excel file in a structured format, mapped to the original sample ID and trace filename. To improve sample classification, statistical averaging of descriptors is performed across all detected peaks, with each peak weighted by its intensity. This yields a representative elution fingerprint for each sample type. The feature matrix serves as the foundation for further analyses, including dimensionality reduction, clustering, and supervised classification.

To support transparency and quality assurance, the pipeline also generates diagnostic visualizations for each sample. These plots overlay the raw elution trace, the smoothed baseline, and the fitted elution EMG components, providing a visual summary of the decomposition and fit quality. Such outputs are saved automatically for every sample, facilitating manual review and the identification of potential outliers or fitting anomalies. The entire pipeline is designed for scalability, enabling batch processing of large sample sets, and is easily extensible to incorporate new polymer classes, updated analytical parameters, or evolving experimental protocols (SI, Figure S2 and Table S3).

### 2.3. Data Augmentation and Visualization

To overcome the significant class imbalance in the original dataset, primarily due to the uneven distribution of samples across different PO (sub-)classes, a targeted oversampling strategy was implemented. This step was critical to prevent statistical and machine learning models from being biased toward the most represented (sub-)classes, thereby improving the robustness, generalizability, and predictive accuracy of the classification tasks. The core of the strategy relied on data augmentation techniques designed specifically for the elution peak domain, ensuring the generation of realistic and chemically meaningful synthetic examples that could enrich the feature space without introducing noise or distortion.

Data augmentation was performed using the SMOTE (Synthetic Minority Over-sampling Technique) algorithm [30] via a custom Python-based script that operates directly on the parametric descriptors of the EMG functions (Equation (1)) previously obtained from the elution peak fitting routine. For each PO (sub-)class, synthetic elution traces were created by perturbing the EMG parameters, namely amplitude, center, width, and decay constant, within empirically derived bounds. These ranges were carefully selected based on observed intra-class variability, ensuring that the generated traces remained faithful to the physical and chemical characteristics typical of each PO (sub-)class. The aim was to simulate natural variability found within the same (sub-)class while preserving class-specific fingerprint features related to elution behavior.

Each synthetic trace was reconstructed as a linear combination of modified EMG components and normalized in the same manner as the original traces, preserving comparability across the dataset. Once generated, statistical averaging was again performed on the descriptors of the synthetic peaks to derive class-specific fingerprints, allowing these new data points to be seamlessly integrated into the analytical pipeline. The number of synthetic instances per class was selected to approximately equalize the sample count across all PO (sub-)classes in Table S1, producing a more balanced dataset suited for training supervised learning models.

In parallel, a set of synthetic “negative” traces was also generated to enrich the model with representative counterexamples. Rather than simulating only baseline or noise, these samples were created by randomly sampling across the entire multidimensional feature space defined by the extracted EMG parameters. As such, they span a wide range of plausible values, including regions that may overlap with polymer-containing signals. This approach ensures that the model learns to identify polymer-specific patterns robustly, rather than simply detecting deviations from a baseline, thereby reducing the risk of overfitting and improving generalization to ambiguous or borderline cases.

To further guide feature selection for classification, the augmented dataset was explored through a series of diagnostic scatterplots, which mapped key EMG-averaged parameters against the elution peak position (SI, Figure S3). Parameters such as total peak area, total amplitude, average variance, methyl per thousand carbons, and intrinsic viscosity at the elution peak were plotted to identify separable regions in the feature space. These visual tools provided insight into how distinct polymer (sub-)classes distribute across the elution profile and helped to prioritize the most discriminative descriptors for machine learning. As a complement, Figure S4 reports the same parameter scatterplots for the synthetic negative samples, randomly generated across the feature space.

Overall, the augmentation framework significantly improved the coverage and balance of the dataset. By simulating intra-class variability and introducing chemically plausible synthetic data, while maintaining focus on the elution peak features, the approach enabled a more comprehensive representation of PO (sub-)classes and enhanced the performance and resilience of downstream predictive models.

### 2.4. Machine Learning Models: Generalities

The final step of the workflow involves the application of supervised machine learning models for the recognition and classification of PO monomaterials based on their aCEF profiles and associated analytical parameters. This step was designed to address three distinct prediction tasks, each of which builds upon the previous one: binary classification of PO presence, multiclass classification of PO identity, and regression-based estimation of comonomer composition in case of copolymers. Each model was trained on the augmented dataset created during the earlier stages of data processing, which included both real and synthetic samples. This dataset was constructed from a uniform vector of quantitative descriptors derived from signal fitting, averaging, and metadata extraction.

All machine learning models share a common input representation consisting of analytical descriptors extracted from the aCEF signal and the corresponding EMG fitting parameters. These features include:

- Total normalized area of all EMG peaks;
- Average EMG peaks position;
- Total peak amplitude summed across all EMGs;
- Average variance of the EMGs;
- Average methyl per 1000 carbons value at the peak positions;
- Average intrinsic viscosity at the peak positions.

Each sample, whether experimental or synthetic, was represented as a single vector containing these six features, ensuring full compatibility with standard classifiers and regressors available in scikit-learn. Additionally, each sample was pre-labeled with the corresponding target outputs required for the specific prediction task. For classification tasks, the target labels were either binary or multilabel, depending on the task type. In the case of copolymers, for regression tasks, the target was comonomer content (in mol%), computed from sample metadata or assigned during synthetic generation. The final dataset was split into training (80%) and testing (20%) subsets using a fixed random seed to ensure reproducibility. Although stratified sampling was not explicitly applied, the dataset had been pre-balanced through data augmentation, resulting in approximately uniform class distributions in both subsets. Class proportions were verified post-split to confirm the absence of significant imbalances.

The dataset used for training and testing the machine learning models consisted of a total of 142 samples per polyolefin (PO) class, combining both real and synthetically augmented data. Additionally, a set of 142 synthetic “no-polymer” samples was created to simulate baseline conditions, bringing the overall dataset size to 1278 samples. The choice of 142 samples per class was guided by practical and statistical considerations for Random Forest classifiers [31]. According to empirical guidelines in machine learning literature, although not explicitly stated as a rule, a sample size of approximately 20–30 times the number of features per class is generally considered a reasonable lower bound for stable model performance. Given the limited number of input features in the present work, a total of over 1200 polymer-positive samples provides a sufficiently large and diverse dataset to avoid overfitting, ensure meaningful model training, and support generalization.

### 2.5. Binary, Classification, and Regression Models

The first predictive model addresses the binary task of determining whether a given sample is a true polymeric material. This classifier was trained on a dataset composed of both real polymer samples and synthetic negative controls, which were generated during the previous step using uniformly sampled baseline parameters with Gaussian noise. The model was implemented using a Random Forest classifier [31,32], with hyperparameters optimized via grid search cross-validation. The output of the model is a probability score ranging from 0 to 1, indicating the likelihood of polymer presence. To facilitate practical use, thresholds were defined to categorize samples into two binary categories:

- “No polymer detected” (score < 0.7);
- “Polymer detected” (score ≥ 0.7).

These cutoffs were determined empirically by analyzing the precision–recall curve, optimizing the balance between sensitivity and specificity to minimize false positives and false negatives (SI, Figure S5).

For samples classified as polymer-positive (score ≥ 0.7 in the binary classification), a second predictive model was implemented to identify the specific polymer type(s) present. Although this model was initially applied to the classification of PO monomaterials, it was designed with future applications involving PO mixtures in mind. Therefore, in light of the possibility of multiple PO components in a given multimaterial sample (e.g., iPP + EPR, or LDPE + LLDPE), this task was framed as a multilabel classification problem. The model was built using a MultiOutputClassifier [33] wrapper around a RandomForestClassifier, which enabled independent binary classifiers to be trained for each polymer class present in the dataset. Each classifier outputs the probability of presence for its respective polymer, allowing the model to predict multiple coexisting polymer classes without assuming mutual exclusivity. To interpret these probabilities, the following thresholds were applied:

- Polymer not present (score < 0.3);
- Polymer present with low confidence (score between 0.3 and 0.5);
- Polymer present with moderate confidence (score between 0.5 and 0.8);
- Polymer present with high confidence (score > 0.8)

The polymer classification results are stored in a structured Excel file, where each row corresponds to a real or synthetic sample. For each sample, the file includes the outcome of the binary classification, the actual polymer class, and the model’s interpretation, which lists all detected polymer classes with probabilities above a 0.3 threshold. It also reports the most likely polymer identified by the model, its associated confidence level, and the probability estimates for the presence of each polymer in the material.

The adoption of a two-stage approach (first detecting the presence of a polymer, then classifying its type) was aimed at improving both performance and interpretability. The presence detection step treats the “no polymer” condition as a distinct binary task, reducing false positives and preventing forced classifications when no meaningful signal is detected. Separating the tasks allows for each model to specialize, facilitates threshold optimization, and offers greater modularity and transparency for future refinements.

The third and final model was designed to estimate the composition of samples classified as copolymers, namely LLDPE, raco-PP, and EPR. This task was approached using regression modeling with a RandomForestRegressor [31]. As with the previous models, the same set of six analytical descriptors served as the input features. The regressor was trained on both real and synthetic data, with the target output being the known comonomer content in mol%, in the ranges of commercial relevance indicated in Table S1. The predictions were saved alongside the classifier outputs, and for each sample, both the real and predicted copolymer composition were added to the structured Excel file (SI, Table S4). This allows for users to cross-reference the qualitative presence/absence information with the quantitative composition estimates.

Specifically, the regression model was applied exclusively to samples that had been correctly classified in terms of copolymer (sub-)class, ensuring that composition estimates were only derived from confidently identified materials.

Random Forest (RF) models were selected for both binary/classification and regression tasks due to their robustness, ease of use, and strong performance on structured datasets such as the one used in this study. RF classifiers are particularly well-suited for scenarios involving a limited number of features and relatively small sample sizes, as in our case. They effectively capture nonlinear patterns, are robust to noise, and require minimal hyperparameter tuning [31].

Importantly, Random Forests are straightforward to interpret and analyze, for example, through feature importance scores, and are easy to integrate into modular and scalable pipelines. This was especially beneficial in our two-stage architecture, where separate models were used for presence detection and polymer classification. For regression, Random Forests can capture complex, nonlinear relationships between spectral features and polymer composition without relying on rigid assumptions about the underlying data distribution.

## 3. Results and Discussion

### 3.1. Binary and Multiclass Classification—Polymer Presence and Type

**Figure 2:**
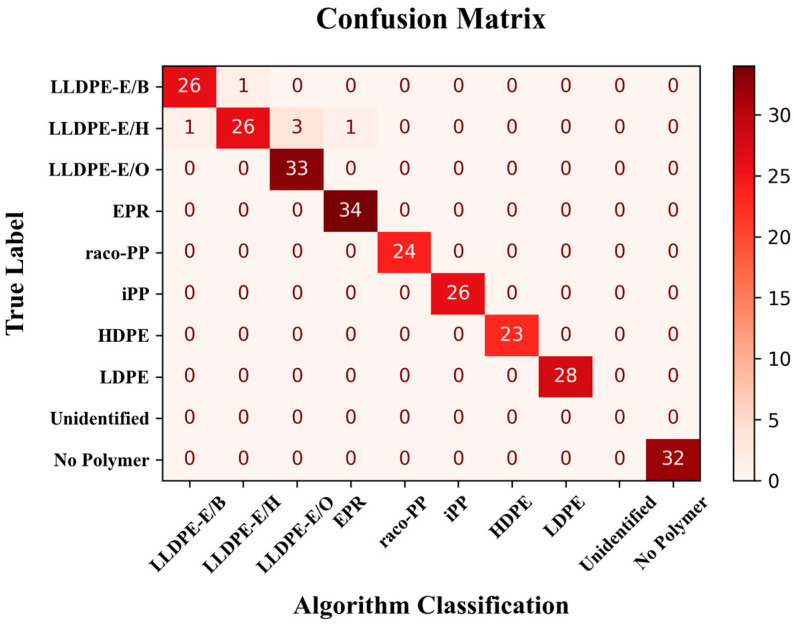
Confusion matrix displaying the performance of the binary presence/absence and polymer-type classification models.

The performance of the developed machine learning pipeline, which integrates a binary classifier for polymer presence detection and a multi-label classifier for polymer-type (PO (sub-)class) identification, is summarized in the confusion matrix reported in Figure 2. For the binary classification task (polymer presence vs. absence), the model was evaluated using an identification threshold of 0.7 on the output probabilities. Under this condition, the classifier demonstrated perfect performance on the test set, achieving 100% precision and 100% recall. All 32 “No Polymer” samples were correctly classified, with no instances of false positives or false negatives. This level of accuracy confirms the robustness of the model in discriminating samples containing polymers from empty or irrelevant signals, which is a crucial step in automating the screening of post-consumer or mixed-material samples.

The polymer-type classification model, in turn, demonstrated excellent performance across the main polymer classes included in the dataset. All samples belonging to the HDPE, LDPE, iPP, raco-PP, and EPR classes were correctly classified (with only one misassignment (false positive) in the EPR case. Some minor limitations were observed in the classification of LLDPE samples, most notably within the LLDPE-E/H sub-class, due to an inherent variability; as a matter of fact, the fingerprint parameter scatterplots in Figure S5 displayed substantial dispersion across nearly all descriptors for reasons that still need to be clarified.

Notably, the system yielded no “Unidentified” outputs: in all cases where the binary model identified the presence of at least one polymer component, the classification model was able to provide a corresponding polymer identity. This outcome confirms the internal consistency between the two stages of the model and suggests that the classification model is well-calibrated with respect to the presence predictions.

To further assess model performance, classification results were grouped according to the confidence level associated with each prediction, based on the maximum class probability returned by the classifier. Table 1 summarizes the number of samples and the corresponding classification accuracy across four confidence intervals.

As shown in Table 1, the majority of classified samples (198 out of 258, approximately 77%) fell within the high-confidence category, where the accuracy reached 100%. Only a limited number of cases (approximately 11%) fell within the low-confidence or moderate-confidence categories, where misclassifications were more likely to occur.

These results demonstrate that confidence filtering may serve as a practical and reliable post-processing step, allowing analysts to tailor the balance between sensitivity and precision depending on the application. Specifically, filtering based on prediction confidence offers a pathway to mitigate uncertainties associated with certain ethylene-rich polymers, without discarding correctly classified high-confidence predictions. This approach is especially valuable for screening large datasets or automated pipelines, where interpretability and trust in the output are critical.

### 3.2. Regression—Composition Estimation

**Figure 3:**
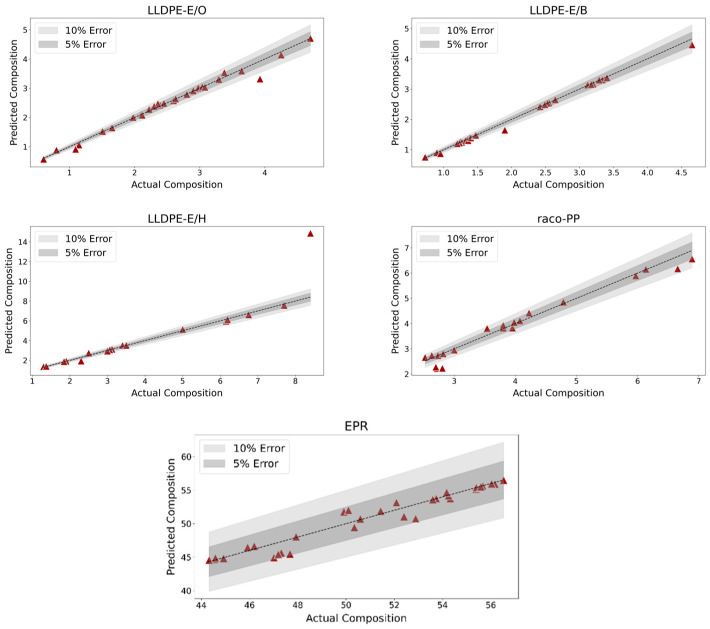
Scatterplot showing the predicted versus actual comonomer content for all correctly classified copolymer samples. The plot includes 5% and 10% relative error bands, indicating the accuracy of the regression model.

The performance of the regression model is illustrated in Figure 3, which displays the predicted versus actual comonomer content for all correctly classified LLDPE-E/B, LLDPE-E/H, LLDPE-E/O, raco-PP and EPR samples. To facilitate interpretation, the plot includes relative error bands at ±5% and ±10%, which serve as visual benchmarks for assessing prediction accuracy.

The distribution of points within these bands reveals the strong agreement between the predicted and actual values across most PO samples, supporting the overall reliability of the model’s quantitative estimates. The plot is particularly useful in highlighting both the tight clustering of data points around the parity line and the rare deviations from expected values. One notable exception is an outlier within the LLDPE-E/H class, which falls significantly outside the confidence intervals and contributes disproportionately to the lower overall performance of the model for such PO sub-classes.

A more detailed breakdown of the regression performance is provided in Table 2, which reports the coefficient of determination (R^2^) for each polymer class, stratified by the confidence level of the classification.

As the table shows, the regression model performed exceptionally well across most PO (sub-)classes, with R^2^ values approaching or equal to 1.00 for LLDPE-E/B, LLDPE-E/O, Raco-PP, and EPR. The model for LLDPE-E/O is particularly noteworthy for its robustness: it maintained excellent accuracy across all confidence levels, including moderate and low-confidence classifications. The lower overall R^2^ observed for the LLDPE-E/H class (0.52) is primarily due to one single outlier sample with a substantial prediction error, also observed in the scatterplot. Importantly, the model achieves a perfect fit (R^2^ = 1.00) for high-confidence LLDPE-E/H samples, indicating that the drop in total performance is not systematic, but rather the result of isolated variability.

To evaluate the consistency and generalizability of the regression model, the coefficient of determination (R^2^) was also calculated on the training set. The R^2^ values obtained for each polymer class in the training set, particularly for predictions associated with high confidence levels, were comparable to those observed in the test set. This consistency confirms the model’s stability and suggests minimal overfitting. Detailed R^2^ values by polymer class and confidence level in the training set are reported in Table 3.

These results demonstrate that, when classification is reliable, the regression model can accurately estimate the intrinsic composition of polyolefins. This capability is crucial for downstream tasks such as compositional fingerprinting, quality control, and process optimization in both research and industrial recycling workflows.

In order to better understand the internal decision process of the Random Forest regression model, a feature importance analysis was carried out based on the importance scores provided by the trained estimator.

**Figure 4:**
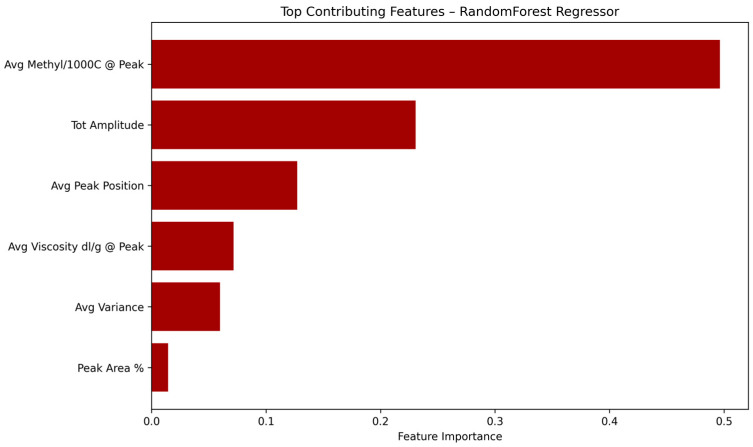
Relative importance of the six input features used in the Random Forest regression model for predicting comonomer content in PO copolymers.

As shown in Figure 4, the most influential feature in predicting comonomer composition was the average methyl per 1000 carbons value at the peak positions, which accounted for nearly 50% of the model’s decision weight. This was followed by the total peak amplitude and average elution peak position, highlighting the importance of chemical structure and elution profile in determining copolymer microstructure. Features related to intrinsic viscosity and elution peak variance were also relevant, though with lower contributions.

## 4. Conclusions

Analytical Crystallization Elution Fractionation (aCEF) is an important technique for the characterization of PO materials. Originally introduced as a faster and more efficient version of Temperature Rising Elution Fractionation (TREF) [25,26], it has progressively gained further applications thanks to the smart utilization of FTIR and dual-capillary viscometry detectors. As a matter of fact, in addition to the distribution of crystallizable sequences in any PO samples, it can also provide semiquantitative information on average molar masses via intrinsic viscosity measurements and, with proper calibrations, on comonomer contents for copolymer samples of known nature.

In this work, we demonstrated that, with the aid of AI methods, it is possible to move further important steps forward. In fact, custom-made machine learning algorithms can be trained on aCEF traces to identify and categorize practically all commercially relevant PO monomaterials *without any prior information*, with average molar masses and comonomer contents for copolymers added as a bonus.

Extension to PO mixtures, including multimaterial waste streams, is a most desirable—albeit most complex too—ultimate objective. In our opinion, integrating automated AI-aided aCEF and ^13^C NMR characterizations is a realistic intermediate step that can already enable higher-value-added solutions in the mechanical recycling of post-consumer PO blends.

## Figures and Tables

**Table 1:** Summary of classification results based on confidence levels, with the number of samples and corresponding accuracy reported for each category.

Category	Samples	Accuracy (%)
Unidentified	0	0
No Polymer	32	100.0
Low Confidence	6	66.7
Moderate Confidence	22	81.8
High Confidence	198	100.0

**Table 2:** Coefficient of determination (R^2^) for each polymer class at different levels of classification confidence for the validation set.

Polymer	R^2^ (Total)	R^2^ (High)	R^2^ (Moderate)	R^2^ (Low)
EPR	0.94	0.94	-	-
LLDPE-E/O	0.98	0.99	1.00	0.97
LLDPE-E/B	1.00	1.00	-	-
LLDPE-E/H	0.52	1.00	−0.15	-
raco-PP	0.97	0.97	-	-

**Table 3:** Coefficient of determination (R^2^) for each polymer class at different levels of classification confidence for the training set.

Polymer	R^2^ (Total)	R^2^ (High)	R^2^ (Moderate)	R^2^ (Low)
EPR	0.99	0.99	-	-
LLDPE-E/O	0.99	0.99	0.98	-
LLDPE-E/B	0.62	0.99	−0.26	-
LLDPE-E/H	0.85	0.92	0.14	-
raco-PP	0.97	0.98	0.82	-

## Data Availability

The original contributions presented in this study are included in the article and/or Supporting Information. Further inquiries can be directed to the corresponding authors.

## Supplementary Materials

The following supporting information can be downloaded at: https://www.mdpi.com/article/10.3390/polym17121597/s1, Table S1. Proposed microstructural categorization of commercial PO monomaterials; Table S2. Structure of the Excel sheet corresponding to a single LDPE sample (as a representative example). The data structure includes the sample ID, polymer class, composition data (for copolymers) retrieved from external metadata, the CEF elution signal, the point-by-point FTIR-calculated value of methyl carbons per 1000 carbons, and the point-by-point value of intrinsic viscosity; Figure S1. Illustration of the iterative EMG fitting process applied to the elution region of a iPP sample (for exemplification purposes); Figure S2. Example of EMG-based elution peak fitting for multiple raco-PP samples (for exemplification purposes). Each plot displays the original CEF trace (black), the detected elution peaks (cross markers), the individual EMG components (colored dashed lines), and the resulting composite fit (solid red line); Table S3. Overview of the Excel file summarizing the extracted EMG fit descriptors for raco-PP samples (for exemplification purposes). Each row represents a sample and includes its aggregated statistical descriptors derived from the fitted crystalline components; Figure S3. Scatterplots of key EMG-derived descriptors across the augmented dataset (excluding synthetic negative samples). Each point represents a PO sample, with colors indicating PO (sub-)classes. Parameters such as total elution peak area, cumulative amplitude, average variance, methyl per thousand carbons, and intrinsic viscosity are plotted against the peak center position; Figure S4. Scatterplots of key parameters for synthetic “No Polymer” samples, randomly distributed across the multidimensional parameter space; Figure S5. Precision–Recall curve for the classification model. Precision represents the percentage of true positives among all instances classified as positive, while recall indicates the percentage of true positives which were classified as positives. A higher precision means lower numbers of false positives, while higher recall means a lower number of false negatives; Table S4. Structure of the Excel file containing the results of the machine learning model for polymer classification and composition estimation. The presence probabilities for each polymer, not reported in the figure, are also included in the file for a detailed interpretation of the results.
