# Supplementary material for: AI-Aided Crystallization Elution Fractionation (CEF) Assessment of Polyolefin Resins
Source: Polymers (Basel). 2025 Jun 7;17(12):1597. doi: 10.3390/polym17121597 (PMC12196792; doi:10.3390/polym17121597)
Supplement: Supplementary file 1 [file polymers-17-01597-s001.zip › polymers-3650608-supplementary.pdf]

Supporting Information

AI-aided Crystallization Elution Fractionation (CEF) assessment of polyolefin resins

Lorenzo Brighel[1,2], Gabriella Maria Lucia Scuotto[1], Giuseppe Antinucci[1,2], Roberta Cipullo[1,2] and Vincenzo Busico[1,2,]*

[1] Department of Chemical Sciences, Federico II University of Naples, via Cinthia, 80126 Napoli, Italy;

[2] DPI, 5600 AX Eindhoven, The Netherlands

[*]Correspondence: busico@unina.it (V.B.)

Table S1. Proposed microstructural categorization of commercial PO monomaterials.

Class	Sub-Class [a]	Nature	Composition [b]
High Density PE (HDPE)	-	Homopolymer	n.a.
Linear Low-Density PE (LLDPE)	E/B	Copolymer	x(B) < 10%
	E/H	Copolymer	x(H) < 10%
	E/O	Copolymer	x(O) < 10%
Low-Density PE (LDPE)	-	Homopolymer	-
Isotactic PP (iPP)	-	Homopolymer	-
raco-PP	-	Copolymer	x(E) < 10%
Ethylene/Propylene Rubber (EPR)	-	Copolymer	40% < x(E) < 60%

[a] E = ethene, P = propene, B = 1-butene, H = 1-hexene, O = 1-octene. [b] Typical commercial range; x(Y) = mole fraction of (co)monomeric unit Y.

	A	B	C	D	E	F
1	**ID**	**Polymer**	**Composition**	**Derivative Norm**	**Methyl/1000C**	**Viscosity dl/g**
2	LDPE 1 - 2 4 1	LDPE	0	0	71.03359985	2.114031553
3				-0.000353904	70.90770721	2.337460756
4				0.000483875	70.7818222	1.248296261
5				0.010293151	70.65593719	0.365828097
6				0.018135672	70.53005219	0.829845786
7				0.010551957	70.40416718	1.29430151
8				0.00236018	70.27828217	0.915152848
9				0.001292931	70.15239716	1.169293404
10				0.001302314	70.02651215	1.194022179

Table S2. Structure of the Excel sheet corresponding to a single LDPE sample (as a representative example). The data structure includes the sample ID, polymer class, composition data (for copolymers) retrieved from external metadata, the CEF elution signal, the point-by-point FTIR-calculated value of methyl carbons per 1000 carbon, and the point-by-point value of intrinsic viscosity.

The whole dataset is organized into separate Excel files for each sample type. Each file contains individual sheets for every sample, where the sample ID, composition data, and relevant analytical data are stored. This organized structure ensures consistency across the dataset, providing a solid foundation for subsequent stages in the workflow, including peak detection, signal fitting, and machine learning applications.

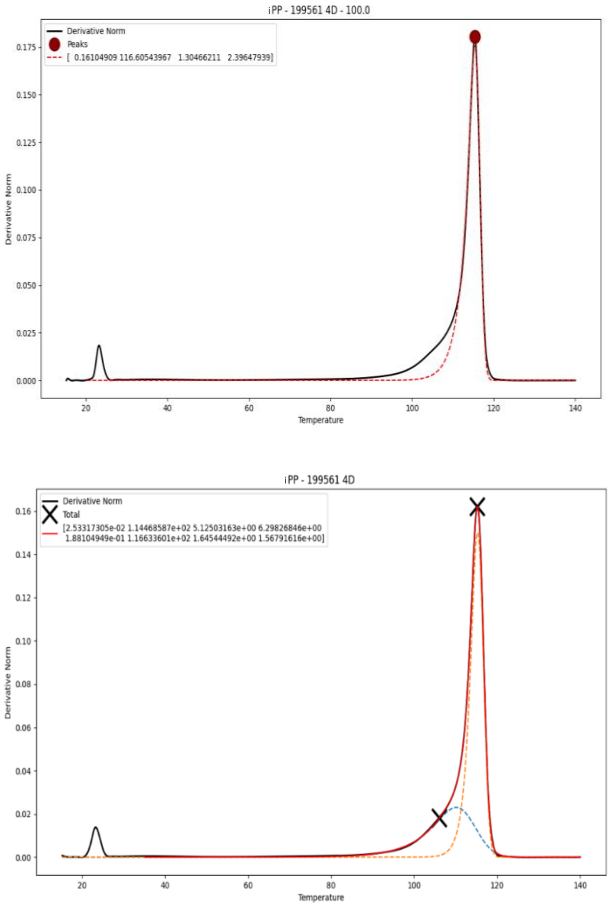

Figure S1. Illustration of the iterative EMG fitting process applied to the elution region of a iPP sample (for exemplification purposes).

The single EMG fit captures the general peak shape but underestimates key features, resulting in residuals and suboptimal coverage of the elution peak area. Through iterative refinement, additional EMG components are introduced selectively, leading to an improved composite fit that accurately reconstructs the asymmetry and fine structure of the peak.

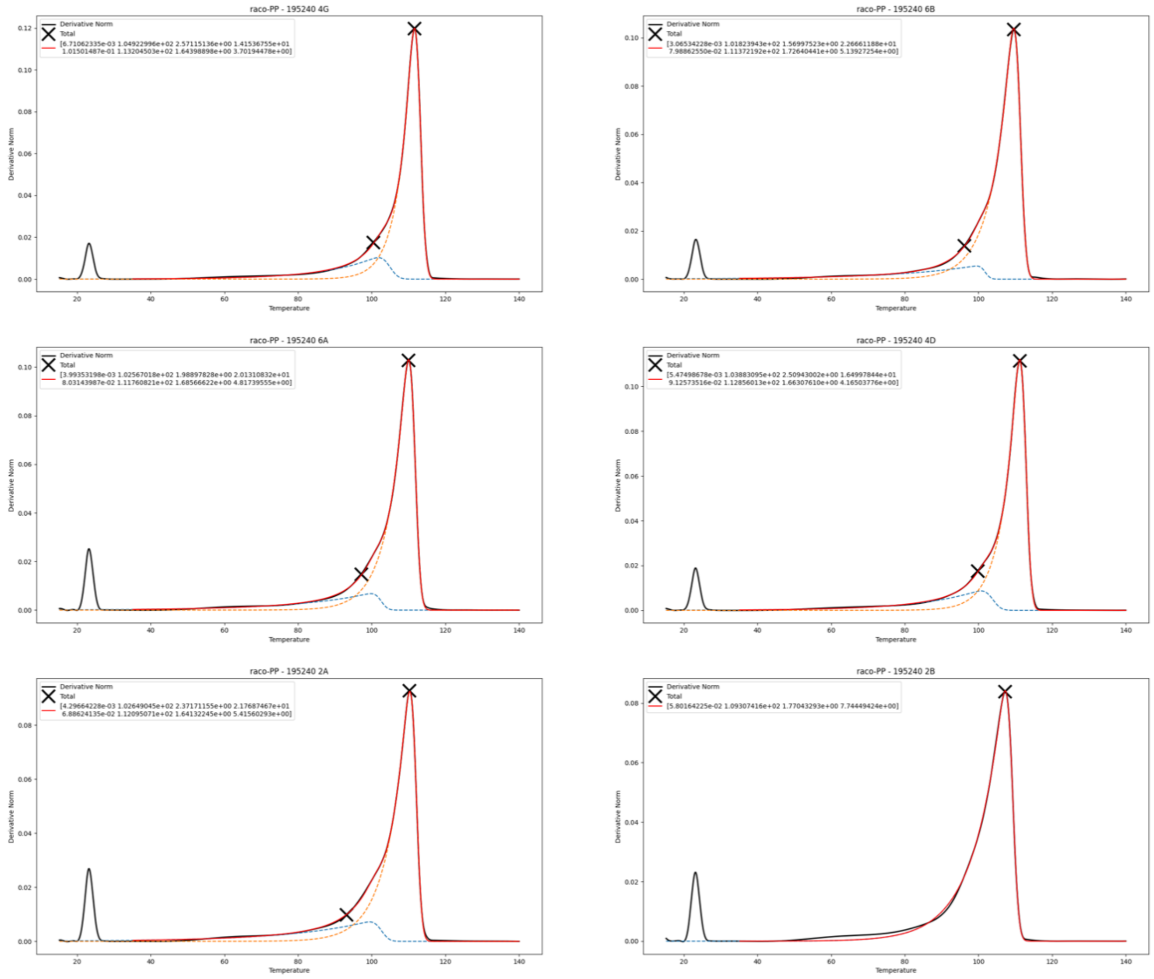

Figure S2. Example of EMG-based elution peak fitting for multiple raco-PP samples (for exemplification purposes). Each plot displays the original CEF trace (black), the detected elution peaks (cross markers), the individual EMG components (colored dashed lines), and the resulting composite fit (solid red line).

	A	B	C	D	E	F	G	H	I	J	K	L
1	ID	Polymer	Composition	#Peaks	Accuracy	Peak Area %	Tot Amplitude	Avg Peak Position	Avg Variance	Avg Tau	Avg Methyl/1000C @ Peak	Avg Viscosity dl/g @ Peak
2	195240 4B	raco-PP	1.8	2	0.99	84.15	0.12	112.23	1.84	5.26	332.84	2.19
3	195240 4G	raco-PP	2.3	1	0.90	85.90	0.08	113.34	1.41	5.08	334.03	3.21
4	195240 6B	raco-PP	2.5	1	0.93	89.10	0.07	111.46	1.57	6.15	333.98	2.95
5	195240 4F	raco-PP	2.7	2	0.99	82.45	0.11	111.61	1.84	5.72	333.54	2.13
6	195240 6A	raco-PP	2.8	1	0.92	85.95	0.07	111.87	1.50	6.07	334.16	3.04
7	195240 4D	raco-PP	3	1	0.91	85.73	0.08	112.99	1.45	5.50	333.83	2.87
8	195240 4E	raco-PP	3.05	2	1.00	84.30	0.10	111.54	1.88	6.67	333.49	2.15
9	195240 2A	raco-PP	3.3	1	0.92	85.38	0.06	112.23	1.43	7.16	334.96	2.21
10	195240 2B	raco-PP	4	1	0.96	90.06	0.06	109.31	1.77	7.74	334.16	3.00
11	195240 6C	raco-PP	4	1	0.95	86.08	0.05	110.42	1.66	8.37	335.49	2.96
12	195240 2D	raco-PP	4.5	1	0.94	83.45	0.05	111.27	1.50	8.71	336.45	2.55
13	195240 6E	raco-PP	4.9	1	0.96	87.14	0.05	110.15	1.68	8.61	334.55	2.43
14	195240 2E	raco-PP	5	1	0.93	80.53	0.05	112.08	1.41	8.71	335.10	2.12
15	195240 6G	raco-PP	6.7	1	0.97	80.49	0.04	110.07	1.72	10.41	333.86	2.71
16	195240 2H	raco-PP	7.1	1	1.00	67.25	0.03	109.27	1.86	12.96	334.96	2.28

Table S3. Overview of the Excel file summarizing the extracted EMG fit descriptors for raco-PP samples (for exemplification purposes). Each row represents a sample and includes its aggregated statistical descriptors derived from the fitted crystalline components.

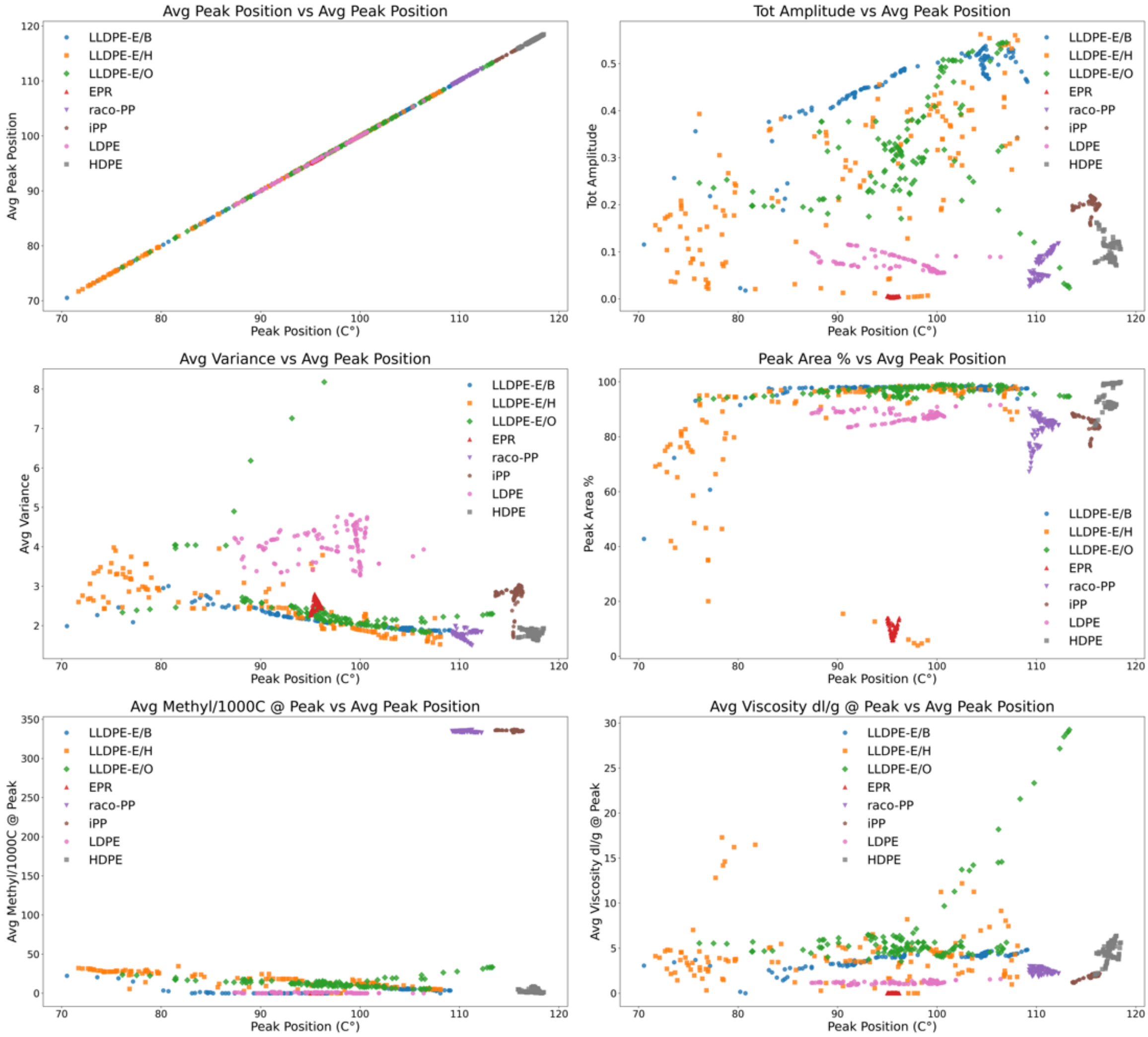

Figure S3. Scatterplots of key EMG-derived descriptors across the augmented dataset (excluding synthetic negative samples). Each point represents a PO sample, with colors indicating PO (sub-)classes. Parameters such as total elution peak area, cumulative amplitude, average variance, methyl per thousand carbons, and intrinsic viscosity are plotted against the peak center position.

.

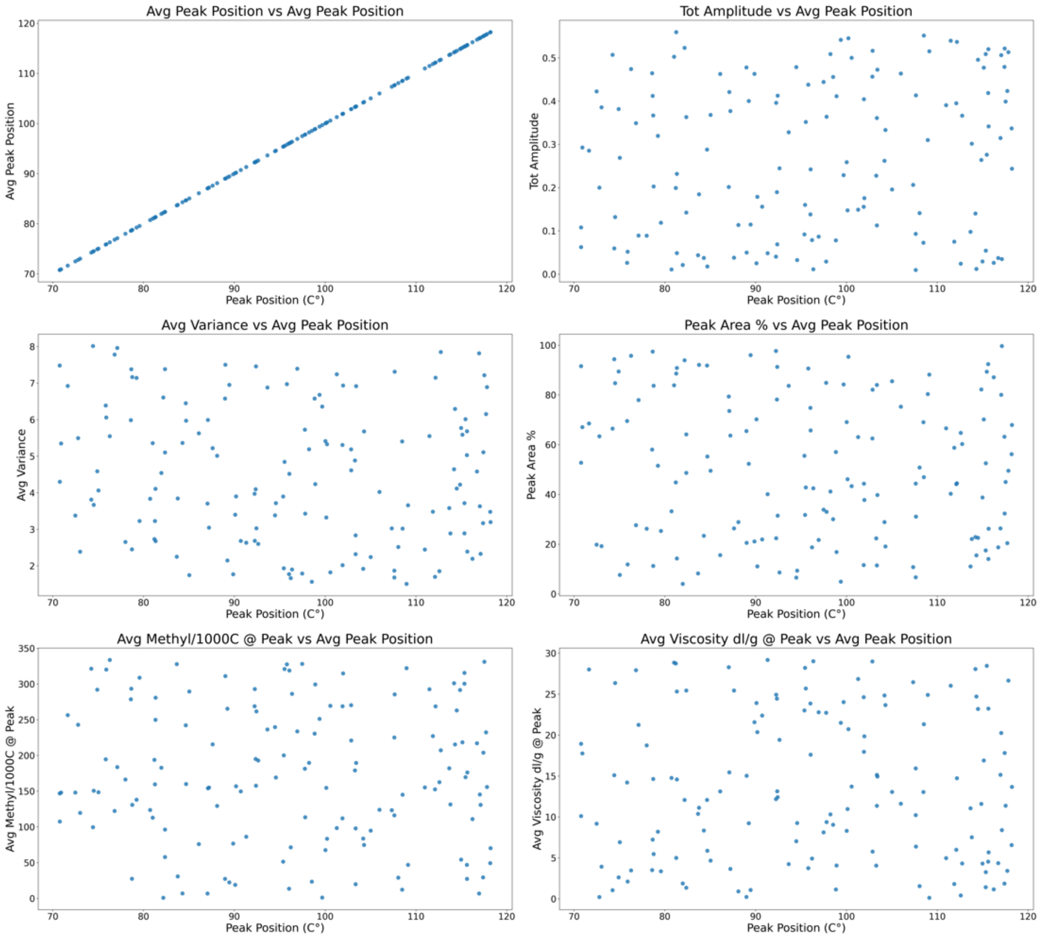

Figure S4. Scatterplots of key parameters for synthetic “No Polymer” samples, randomly distributed across the multidimensional parameter space.

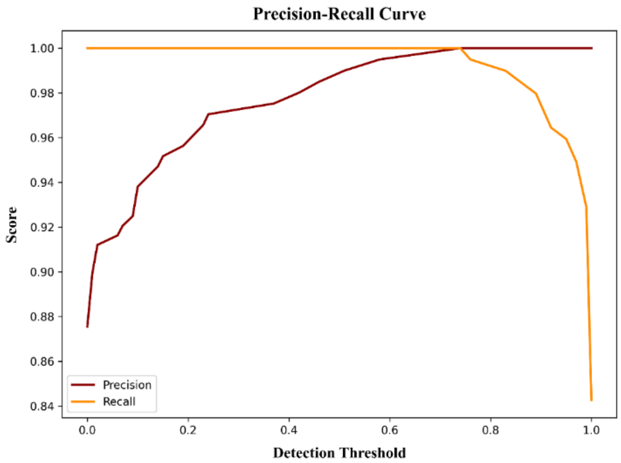

Figure S5. Precision-Recall curve for the classification model. Precision represents the percentage of true positives among all instances classified as positive, while recall indicates the percentage of true positives which were classified as positives. A higher precision means lower numbers of false positives, while higher recall means lower number of false negatives.

	A	B	C	D	E	F	G
1	**Presence Prob**	**True Polymer**	**Interpretation**	**Pred Polymer**	**Confidence**	**True Composition**	**Pred Composition**
2	0.00	No Polymer	No Polymer	No Polymer			
3	1.00	LDPE	LDPE	LDPE	High		
4	1.00	iPP	iPP	iPP	High		
5	0.88	LLDPE-E/H	LLDPE-E/O	LLDPE-E/O	Moderate	7.19	5.51
6	0.09	No Polymer	No Polymer	No Polymer			
7	1.00	EPR	EPR	EPR	High	54.32	53.76
8	1.00	iPP	iPP	iPP	High		
9	0.98	LLDPE-E/O	LLDPE-E/H + E/O	LLDPE-E/O	Moderate	0.80	0.89
10	0.09	No Polymer	No Polymer	No Polymer			
11	1.00	LDPE	LDPE	LDPE	High		
12	0.98	LLDPE-E/B	LLDPE-E/H	LLDPE-E/H	Low	6.68	19.36
13	1.00	iPP	iPP	iPP	High		
14	1.00	LDPE	LDPE	LDPE	High		
15	1.00	EPR	EPR	EPR	High	53.73	53.75
16	1.00	raco-PP	raco-PP	raco-PP	High	4.07	4.11
17	1.00	LLDPE-E/O	LLDPE-E/O	LLDPE-E/O	High	2.90	2.92
18	0.99	LLDPE-E/H	LLDPE-E/H	LLDPE-E/H	Moderate	2.30	1.94
19	0.90	LLDPE-E/O	LLDPE-E/O	LLDPE-E/O	Moderate	1.15	1.06
20	1.00	raco-PP	raco-PP	raco-PP	High	4.07	4.11

Table S4. Structure of the Excel file containing the results of the machine learning model for polymer classification and composition estimation. The presence probabilities for each polymer, not reported in the figure, are also included in the file for a detailed interpretation of the results.
